# Professor Charcot

**Published:** 1887-12

**Authors:** 


### PROFESSOR CHARCOT.

A correspondent of the *Canada Medical Record* remarks of Charcot: Have you ever seen him? Of medium height but most commanding presence; his long hair drawn back from his massive forehead and hanging down his neck; his head poised high and bringing into strong prominence his aquiline nose; his eagle eyes, which pierce through yours so that he seems to read your very soul, but which you cannot look beyond. No wonder that he can tame the wild maniacs of the Saltpetriere with one magic glance. He calls it hypnotism, this power that he has, and he and many others say that many people might learn to acquire it. But I think it is mesmerism *par et simple;* that incomprehensible power which a great mind has over a weaker one. By it he is able to cure many diseases of defective innervation, of the hysterical class, which are due to weakness or absence of will power, and which he supplies for them like the "Great Physician," commanding the paralyzed to take up their bed and walk; or to see him step up to another tortured with ceaseless movements, which are at once arrested by a single look. What a charming lecturer! He does not call them lectures or clinics, but conferences. We all sit around him, leaving a little open space between him and the patients about whom he is speaking, and he just talks away as if he was recounting reminiscences of the past; now a case, now an anecdote, now a theory and now a fact, but every one of them directly to the point. As you listen, you, too, become infatuated with him, and feel that you must do like the poor maniacs, and cast yourself in humble submission at his feet. Two years ago I prepared a paper on a case of genuine scleroderma under my care at the Children's Hospital in London. Charcot had such a case, but not nearly so marked. But how he described it! All that I had discovered about it in six months of research he gave forth in polished and familiar terms.

